# The Impact of the First Peak of the COVID-19 Pandemic on a Paediatric Ophthalmology Service in the United Kingdom: Experience from Alder Hey Children’s Hospital

**DOI:** 10.22599/bioj.164

**Published:** 2021-03-25

**Authors:** Megan Wood, Judith Gray, Ankur Raj, Jose Gonzalez-Martin, Damien C.M. Yeo

**Affiliations:** 1 Alder Hey Children’s NHS Foundation Trust, GB

**Keywords:** paediatric ophthalmology, COVID-19, pandemic, service, NHS

## Abstract

**Objective:**

The COVID-19 pandemic has led to significant service loss across the NHS, and ophthalmology is one of the greatest affected specialties. We attempt to quantify the impact of the first peak of the COVID-19 pandemic on a paediatric ophthalmology unit in a children’s hospital in the United Kingdom (UK) and report lessons learnt to aid in the recovery of the service.

**Methods and Analysis:**

Two eight-week periods of clinical activity were compared; one during the first UK peak of the COVID-19 pandemic and the other during a similar period the previous year. Four areas of clinical activity were included in the study: outpatient clinic appointments, theatre activity, outpatient referrals to ophthalmology and ward reviews. Appointment data was collected from departmental databases.

**Results:**

During the first peak of the pandemic, outpatient clinic appointments were reduced by 87.2%, ophthalmic surgery by 90.9%, outpatient referrals to ophthalmology by 50.2% and ward reviews by 50%. The number of actual cancelled appointments was 1377, of which 6.8% were triaged as suitable for teleophthalmology.

**Conclusion:**

The COVID-19 pandemic has dramatically restricted clinical activity in the ophthalmology service. Paediatric ophthalmology is vulnerable to capacity issues and the consequences of delayed or cancelled appointments. Departments must adapt quickly and maximise capacity to help reduce the backlog and treat patients effectively and safely. Solutions such as teleophthalmology have potential although can be difficult in the paediatric population.

## Introduction

During the COVID-19 pandemic in 2020, ophthalmology departments across the United Kingdom (UK) and the rest of the world were forced to remodel their practice to help protect patients and staff from the spread of the virus. The Royal College of Ophthalmologists (RCOphth) issued guidance on how to approach ophthalmic care during the pandemic, balancing the risks of viral spread with progression of ophthalmic disease and the threat of vision loss (RCOphth, 2020b). Departments were initially advised to postpone all routine surgery and face-to-face outpatient appointments unless patients were at high risk of harm from delay. Reports from USA demonstrated that ophthalmology was the specialty with the largest decrease in outpatient visits, reducing by 79–81% in April/May 2020 (Mehrotra et al. 2020) (News 2020).

Following the first peak, in May 2020, a cautious reopening of clinical activity was advised. However, capacity remained limited due to measures such as social distancing and infection control procedures (RCOphth, 2020c). The ophthalmology service in the UK was already under strain prior to the pandemic, with the majority of units dependent upon waiting list initiatives (RCOphth, 2018). Since the first peak the UK has had two further lockdowns and a second peak of infections over winter 2020/21. Now it is faced with an indefinite period of reduced activity, with a growing backlog of patients.

We attempt to quantify the impact of the first peak of the COVID-19 pandemic on our service and discuss the issues that will impact our recovery.

## Materials and Methods

We compared two eight-week periods of clinical activity at Alder Hey Children’s Hospital (AHCH) Ophthalmology Department; one period at the first peak of the COVID-19 pandemic (23^rd^ March 2020–15^th^ May 2020), during the maximum disruption to the service, and the same 8-week period in the previous year (25^th^ March 2019–17^th^ May 2019). Data was collected in four areas of our department: outpatient clinics, theatre activity, ward reviews and referrals to ophthalmology. The number and type of appointments, attendance rate and outpatient referral information were collected from the hospital’s clinic databases. Outpatient clinic appointments include ophthalmology, optometry, orthoptics and electrophysiology clinics. The number of patients undergoing ophthalmic surgery and the procedure type was obtained from theatre records. The number of ward reviews were collected from an electronic clinic diary.

## Results

A significant reduction in clinical activity was seen in all areas of the paediatric ophthalmology service during the first peak of the COVID-19 Pandemic compared to the previous year (***Table 1***).

**Table 1:** Service loss in Paediatric Ophthalmology at AHCH During the First Peak of the COVID-19 Pandemic.


	PRE-COVID	COVID 1^ST^ PEAK	ACTIVITY RATE (%)

	(25/03/2019–17/05/2019)	(23/03/2020–15/05/2020)	

**Outpatient clinic appointments**	2377	304	–87.2%

**Ophthalmic surgery**	66	6	–90.9%

**Outpatient referrals**	285	142	–50.2%

**Ward reviews**	12	6	–50.0%

**Total activity**	2740	458	–83.3%


Over the eight-week study period during the first peak of the COVID-19 pandemic, the number of outpatient appointments reduced significantly compared to the same eight-week period in 2019. The total number of actual existing appointments that were cancelled or postponed during the COVID-19 period was 1377. The cancelled appointments were triaged into 3 risk categories; in accordance with the RCOPhth guidance and trust policy (RCOphth 2020a). For example, urgent face-to-face reviews included patients with significant change in vision, acute onset diplopia and leukocoria. The triage process was conducted by doctors, orthoptists and optometrists with a range of experience. This would have led to variability in the triaging process, despite the use of guidelines. However, multiple clinicians were necessary due to the high volume of patients involved. 7.8% were considered high risk (not for cancellation), 50.8% were medium risk (could be deferred up to 3 months) and 41.4% were deemed low risk (could be deferred up to 6 months). Only 90 patients (6.8%) of all cancelled appointments were triaged as appropriate for teleophthalmology. The DNA (did not attend) rates were similar; 11.4% during the pandemic compared to 13.3% in the previous year.

Only 6 paediatric ophthalmic operations were performed during the pandemic period (1 examination under anaesthesia (EUA), 2 intraocular and 3 retinal laser procedures). For comparison, 66 operations were performed in the same period in 2019 (11 EUA, 28 strabismus, 8 intraocular, 13 oculoplastics, 4 retinal lasers and 2 others). This reflected a significant decrease in paediatric ophthalmic surgery between the two time periods. The number of actual booked operations cancelled or postponed during the eight-week COVID-19 period was 45 (28 strabismus, 4 EUA, 10 oculoplastics, 1 intraocular and 2 retinal lasers). Each case was individually assessed to determine whether postponement was feasible. Surgical cases were postponed based on the discretion of the consultant paediatric ophthalmologists—balancing the risk of vision loss against the risk of viral spread to the patient and staff and also taking into account the vastly reduced theatre capacity across all departments.

Overall, there was a reduction in the number of referrals to the ophthalmology service in the COVID-19 year compared to the previous year (***Table 2***). This reduction was in all categories, including from internal/external consultants, allied health care professionals (including optometrists), general practitioners and the emergency department. The largest fall in referrals was from the emergency department.

**Table 2:** Change in Referrals to Paediatric Ophthalmology at AHCH During the First Peak of the COVID-19 Pandemic.


REFERRER	PRE-COVID	COVID 1^ST^ PEAK	REFERRAL RATE (%)

	(25/03/2019–17/05/2019)	(23/03/2020–15/05/2020)	

**General Practitioner**	168	82	–51.2%

**Internal Consultant**	63	32	–49.2%

**External Consultant**	21	11	–47.6%

**Emergency Department**	15	5	–66.7%

**Allied Healthcare Professional**	15	9	–40.0%

**Unknown**	3	3	0.0%

**Total Referrals**	285	142	–50.2%


The number of ward reviews reduced by 50% (from 12 ward reviews in 2019 to 6 in 2020). This is likely due to the reduced number of admissions to hospital during the first wave of the pandemic.

## Discussion

The COVID-19 pandemic has led to a dramatic service loss in ophthalmology departments worldwide.

Attempts to quantify service loss considering only the number of actual outpatient appointment and surgeries cancelled would lead to a significant underestimation, as evidenced by the figures we collected. At the height of the first peak, 304 outpatient appointments took place and 1377 were cancelled. If these appointments were not cancelled, the total number of theoretical consultations would have been 1681, which is 696 (29%) less than the same period in 2019 suggesting that counting cancellations alone does not accurately reflect the decrease in activity.

This reduction is partly explained by the drop in referrals to ophthalmology, for example Moorfields Eye Hospital reported a 62% reduction in cases presenting requiring retinal detachment surgery (Wickham et al. 2020). ***Table 2*** similarly demonstrates the reduced referrals experienced at AHCH. However, figures also suggest that there is a cohort of patients that are not presenting to a clinician (General Practitioner, Optometrist or Emergency Department) when they have an ophthalmological problem, likely due to fears regarding viral spread and overburdening the NHS. There is concern that those that are delaying presentation may have more advanced disease and therefore may have a poorer prognosis, requiring more intensive or frequent treatments later in their care.

DNA rates in the COVID-19 period remained the same despite limited appointments and a reminder telephone call to the patients. We were unable to fully understand the reasons for this but postulate that it may be due to fears concerning viral spread.

According to the 2019 data we should be expecting a back log of around 2000 patients still awaiting outpatient appointments from the eight-week first peak of COVID-19 period alone. Estimating the ongoing increasing backlog is difficult due to the unpredictable nature of a pandemic, and the indefinite period of ongoing constraints advised by the Government and the NHS. After the first peak, clinical capacity slowly increased although it was still necessary to work at a reduced capability, compounding the backlog of cancellations. Winter 2020/2021 has seen a dramatic increase in COVID-19 cases, in part due to the emergence of new strains of the virus, leading to a repeat of harsher restrictions similar to the first peak (Kirby 2021). The vaccines offer hope of a return to normality; however the future remains unpredictable, especially considering the mutation of the virus (Polack et al. 2020; Voysey et al. 2021). Ophthalmology departments are faced with the task of attempting to maximise capacity whilst maintaining a safe environment for staff and patients.

The main challenge and restriction on capacity in outpatients was the requirement to install rigorous infection control policies to prevent viral spread. Hospital policy is based on national guidelines, such as the operating framework providing by the NHS (NHS 2020). Social distancing restricted waiting room capacity and the number of staff in clinic. Staff numbers were reduced due to requirements to self-isolate and vulnerable staff shielding. Staff were required to work from home where possible. Appointments had to be extended to allow for more stringent cleaning and increased use of personal protective equipment (PPE). Theatre operating time also increased due to similar infection control measures. Before their operations, patients were required to self-isolate and test negative for COVID. This additional step increased the administrative burden as well as reduced the flexibility in filling theatre lists when patients were cancelled.

During the first peak, the Alder Hey Ophthalmology Department was forced to implement many strategies to adapt to the new circumstances. Previously non-clinical spaces such as meeting rooms were repurposed to allow more space for waiting patients. The clinic hours were extended (for example: 9.00am to 5.00pm shifts were changed to 8.00am to 6.00pm shifts) and additional Saturday clinics were commenced. Appointment times were more widely spaced to reduce numbers in clinic and allow additional time for a more intensive cleaning regime. Patients were posted dilating drops to instil at home, where appropriate, to improve patient flow. The waiting lists for surgery and outpatient appointments have been validated by clinicians, to reduce unnecessary appointments and risk-stratify the remaining patients. Earlier discharge to community optometrists has been encouraged if the clinical picture is likely to be stable. Virtual diagnostic clinics were increased, where patients attend for investigations only, and the results were then remotely assessed by clinicians. Teleophthalmology was expanded, mainly in the form of telephone calls for waiting list stratification but also with video consultations using the Attend Anywhere web-based platform. We have since expanded to include virtual photograph clinics, where parents submit photos of their child’s eyes prior to a telephone consultation. Early feedback has found parents favour this consultation compared to video consultations as it can be difficult to engage some children to examine them during video calls.

Paediatric ophthalmology faces a few unique challenges, as identified by RCOPhth guidelines (RCOphth 2020a). Capacity is more severely limited by social distancing as all patients will attend clinics accompanied by an adult. Although a gamut of teleophthalmology solutions, including remote vision testing, has been trialled and rolled out with moderate degrees of success in adult ophthalmology subspecialties (Bourdon et al. 2020; Gillam et al. 2020; Kang et al. 2020; Kilduff et al. 2020; Mintz et al. 2020; Crossland et al. 2021; Faes et al. 2021), it may be more difficult to adapt these to the paediatric population. Our paper reveals a striking statistic: when triaging cancelled outpatient appointments during the first peak at AHCH, only 6.8% were deemed to be suitable for a telephone or video consultation. Local concerns regarding the use of remote consultations mainly stemmed from patient safety, confidentiality, reliability of particularly orthoptic-based assessments and lack of reliable and functional technology. In a survey of UK orthoptists, the main concerns expressed regarding the adoption of teleophthalmology were ethics, confidentiality and poor technology (Rowe et al. 2020). The British and Irish Orthoptic Society and RCOphth have advised caution with at-home vision testing apps as their reliability is not yet proven (RCOphth and BIOS 2020). We have found poor compliance and engagement with telemedicine, particularly if parents are required to work with unfamiliar software. Painter et al. similarly found only 15% of parents undertook home vision testing when instructed. These factors need to be taken into consideration when innovating technology to aid remote consultations in paediatric ophthalmology.

Timings of appointments can be critical in paediatric ophthalmology, with delays potentially leading to permanent vision loss (for example in amblyopia, cataracts and uveitis) or delayed diagnosis of life-threatening conditions (Epelman 2012). Currently it is too early to assess or quantify the harm caused in our population. However, in an unpublished departmental audit of 711 school referrals from 2019, 583 (82%) children were true positive referrals that required either optical management or further diagnostics. Worryingly, vision screening in school has only been tentatively restarted in March 2021, leading to at least a 12-month delay in these children receiving formal visual assessment. It is therefore important to adapt and innovate to reduce the morbidity and mortality as a result of postponement of non-urgent medical care.

## Conclusion

The COVID-19 pandemic has dramatically restricted clinical activity within our ophthalmology department and in the UK. Our paediatric ophthalmology service has suffered a substantial service loss across all areas. Although clinical activity has increased in line with the NHS’s operating framework, restrictions still apply to prevent the spread of the virus. These restrictions are likely to remain in place in the long term. Services have been forced to adapt to the challenges that limit capacity, such as physical distancing, more rigorous cleaning regimes, and PPE. Our department has employed measures such as increasing the amount of physical clinical space available, using additional community clinics, teleophthalmology, virtual clinics, waiting list validations, extended clinic hours and weekend clinics to maximise capacity. Despite some promising teleophthalmology solutions, the majority of our paediatric ophthalmology patients still require a face-to-face assessment. Rapid innovation and validation of these solutions are required to maximise the number of safe remote appointments. In the face of uncertainty and a growing backlog of patients, the ophthalmology service must adapt quickly, using lessons learned in previous peaks, to continue to provide safe and effective care for its patients.

## References

[1] Bourdon, H, et al. 2020. Teleconsultation in primary ophthalmic emergencies during the COVID-19 lockdown in Paris: Experience with 500 patients in March and April 2020. Journal Francais d’Ophtalmologie (Elsevier Masson SAS), 43(7): 577–585. DOI: 10.1016/j.jfo.2020.05.005 PMC7284250 32564983

[2] Crossland, MD, et al. 2021. Evaluation of a Home-Printable Vision Screening Test for Telemedicine. JAMA Ophthalmology (American Medical Association). DOI: 10.1001/jamaophthalmol.2020.5972 PMC7791401 33410910

[3] Epelman, S. 2012. Preserving vision in retinoblastoma through early detection and intervention. Current Oncology Reports (Springer), 14(2): 213–219. DOI: 10.1007/s11912-012-0226-z 22350438

[4] Faes, L, et al. 2021. False alarms and the positive predictive value of smartphone-based hyperacuity home monitoring for the progression of macular disease: a prospective cohort study. Eye (Basingstoke) (Springer Nature). DOI: 10.1038/s41433-020-01356-2 PMC7790308 33414531

[5] Gillam, M, et al. 2020. Teleophthalmology consultations—how do we keep our patients safe? Eye (Basingstoke) (Springer Nature). DOI: 10.1038/s41433-020-01231-0 PMC7586360 33106612

[6] Kang, S, et al. 2020. Oculoplastic video-based telemedicine consultations: Covid-19 and beyond. Eye (Basingstoke) (Springer Nature), 1193–1195. DOI: 10.1038/s41433-020-0953-6 PMC7216849 32398851

[7] Kilduff, CLS, et al. 2020. Creating the Moorfields’ virtual eye casualty: Video consultations to provide emergency teleophthalmology care during and beyond the COVID-19 pandemic. BMJ Health and Care Informatics (BMJ Publishing Group), 27(3). DOI: 10.1136/bmjhci-2020-100179 PMC7430180 32796085

[8] Kirby, T. 2021. New variant of SARS-CoV-2 in UK causes surge of COVID-19. The Lancet Respiratory Medicine (Elsevier BV). DOI: 10.1016/S2213-2600(21)00005-9 PMC7784534 33417829

[9] Mehrotra, A, et al. 2020. The Impact of the COVID-19 Pandemic on Outpatient Visits: A Rebound Emerges | Commonwealth Fund, To the Point (blog), Commonweath Fund. Available at: https://www.commonwealthfund.org/publications/2020/apr/impact-covid-19-outpatient-visits (Accessed: 27 May 2020).

[10] Mintz, J, et al. 2020. Teleophthalmology for age-related macular degeneration during the COVID-19 pandemic and beyond. Journal of Telemedicine and Telecare (SAGE Publications Ltd). DOI: 10.1177/1357633X20960636 PMC9444820 32990152

[11] News, E. 2020. Analysis: Ophthalmology Lost More Patient Volume Due to COVID-19 Than Any Other Specialty – Eyewire News. Available at: https://eyewire.news/articles/analysis-55-percent-fewer-americans-sought-hospital-care-in-march-april-due-to-covid-19/ (Accessed: 27 May 2020).

[12] NHS. 2020. Operating framework for urgent and planned services in hospital settings during COVID-19. Available at: https://www.england.nhs.uk/coronavirus/wp-content/uploads/sites/52/2020/05/Operating-framework-for-urgent-and-planned-services-within-hospitals.pdf (Accessed: 15 June 2020).

[13] Polack, FP, et al. 2020. Safety and Efficacy of the BNT162b2 mRNA Covid-19 Vaccine. New England Journal of Medicine (Massachusetts Medical Society), 383(27): 2603–2615. DOI: 10.1056/NEJMoa2034577 PMC7745181 33301246

[14] RCOphth. 2018. Workforce Census 2018. Available at: https://www.rcophth.ac.uk/wp-content/uploads/2019/02/RCOphth-Workforce-Census-2018.pdf (Accessed: 15 June 2020).

[15] RCOphth. 2020a. Management plans for children and young people with eye and vision conditions during COVID-19. Available at: https://www.england.nhs.uk/coronavirus/publication/covid-19-prioritisation-within- (Accessed: 18 May 2020).

[16] RCOphth. 2020b. RCOphth: Management of Ophthalmology Services during the Covid pandemic Guiding principles. Available at: https://www.gov.uk/government/collections/coronavirus-covid-19-list-of-guidanceandtheRCOphthwebsiteforupdatedinformationhttps://rcophth.ac.uk/2020/03/covid-19-update-and-resources-for-ophthalmologists/ (Accessed: 26 May 2020).

[17] RCOphth. 2020c. Reopening and redeveloping ophthalmology services during Covid recovery-Interim guidance. Available at: https://www.rcophth.ac.uk/wp-content/uploads/2020/04/Reopening-and-redeveloping-ophthalmology-services-during-Covid-recovery-Interim-guidance-1.pdf (Accessed: 26 May 2020).

[18] RCOphth and BIOS. 2020. The use of at home vision testing apps as an adjunct to telemedicine in children. Available at: https://www.rcophth.ac.uk/wp-content/uploads/2020/06/Telemedicine-for-Paediatric-Services-Rapid-Advice-FINAL-090620.pdf.

[19] Rowe, F, et al. 2020. Orthoptic Services in the UK and Ireland During the COVID-19 Pandemic. British and Irish Orthoptic Journal (White Rose University Press), 16(1): 29. DOI: 10.22599/bioj.153 PMC7510392 32999991

[20] Voysey, M, et al. 2021. Safety and efficacy of the ChAdOx1 nCoV-19 vaccine (AZD1222) against SARS-CoV-2: an interim analysis of four randomised controlled trials in Brazil, South Africa, and the UK. The Lancet (Lancet Publishing Group), 397(10269): 99–111. DOI: 10.1016/S0140-6736(20)32661-1 PMC7723445 33306989

[21] Wickham, L, et al. 2020. The impact of COVID policies on acute ophthalmology services—experiences from Moorfields Eye Hospital NHS Foundation Trust. Eye (Springer US). DOI: 10.1038/s41433-020-0957-2 PMC7220649 32405045

